# On the causes and consequences of the uncoupler-like effects of quercetin and dehydrosilybin in H9c2 cells

**DOI:** 10.1371/journal.pone.0185691

**Published:** 2017-10-04

**Authors:** Aleksey V. Zholobenko, Ange Mouithys-Mickalad, Zdenek Dostal, Didier Serteyn, Martin Modriansky

**Affiliations:** 1 Department of Medical Chemistry & Biochemistry, Faculty of Medicine and Dentistry, Palacky University, Olomouc, Czech Republic; 2 Centre for Oxygen, R&D (CORD), Institut de Chimie, Sart-Tilman, Université de Liège, Liège, Belgium; 3 Faculté de Médecine Vétérinaire, Sart Tilman, Liège, Belgium; Virginia Commonwealth University, UNITED STATES

## Abstract

Quercetin and dehydrosilybin are polyphenols which are known to behave like uncouplers of respiration in isolated mitochondria. Here we investigated whether the effect is conserved in whole cells. Following short term incubation, neither compound uncouples mitochondrial respiration in whole H9c2 cells below 50μM. However, following hypoxia, or long term incubation, leak (state IV with oligomycin) oxygen consumption is increased by quercetin. Both compounds partially protected complex I respiration, but not complex II in H9c2 cells following hypoxia. In a permeabilised H9c2 cell model, the increase in leak respiration caused by quercetin is lowered by increased [ADP] and is increased by adenine nucleotide transporter inhibitor, atractyloside, but not bongkrekic acid. Both quercetin and dehydrosilybin dissipate mitochondrial membrane potential in whole cells. In the case of quercetin, the effect is potentiated *post* hypoxia. Genetically encoded Ca^++^ sensors, targeted to the mitochondria, enabled the use of fluorescence microscopy to show that quercetin decreased mitochondrial [Ca^++^] while dehydrosilybin did not. Likewise, quercetin decreases accumulation of [Ca^++^] in mitochondria following hypoxia. Fluorescent probes were used to show that both compounds decrease plasma membrane potential and increase cytosolic [Ca^++^]. We conclude that the uncoupler-like effects of these polyphenols are attenuated in whole cells compared to isolated mitochondria, but downstream effects are nevertheless apparent. Results suggest that the effect of quercetin observed in whole and permeabilised cells may originate in the mitochondria, while the mechanism of action of cardioprotection by dehydrosilybin may be less dependent on mitochondrial uncoupling than originally thought. Rather, protective effects may originate due to interactions at the plasma membrane.

## Introduction

Quercetin is a common dietary flavonoid with a wide range of biological activities. Addition of a coniferyl moiety via the hydroxyl groups of its B ring yields 2,3-dehydrosilybin, which is found as a minor component of silymarin, the well-known hepatoprotective extract of S*ilybum marianum*. Quercetin has been shown to be protective against ischemia-reperfusion (IR) injury in heart [1,2,3], kidney [4] and brain [5,6] with free radical scavenging, dampening of inflammatory response or interaction with intracellular pathways suggested as possible mechanisms. Dehydrosilybin (DHS) has likewise been shown to be cardioprotective, via an equally nebulous mechanism [7], though uncoupling is thought to be important, at least at the level of mitochondria [8].

Uncoupling of respiration occurs when protons that are pumped out of the mitochondrial matrix by the respiratory chain are allowed to re-enter the mitochondria without the generation of ATP by respiratory complex V. This can occur physiologically, for example, due to the action of uncoupling proteins [9] or transient opening of the mitochondrial permeability transition pore (MPTP) [10]. It can also be induced by protonophores, which permeabilise the inner mitochondrial membrane to protons. Classical protonophores, including 2,4-dinitrophenol (DNP), and carbonyl cyanide-4-(trifluoromethoxy) phenylhydrazone (FCCP), can cause complete uncoupling and hence almost completely abrogate ATP synthesis in mitochondria. This makes them highly toxic. However, there is precedent for suggesting an uncoupling based cardioprotective mechanism, as FCCP has been shown to be cardioprotective at low concentrations in a reactive oxygen species (ROS) dependent manner [11,12]. While those studies did raise certain questions about the exact nature of the process, they do demonstrate that uncoupling by FCCP can lead directly to preconditioning.

A study by Tang *et al*. [2] found quercetin induced preconditioning to be abrogated by the inhibition of Protein Kinase Cε (PKCε) signalling in cardiomyocyte culture. However, PKCε integrates upstream signalling from cytoplasm and feeds into mitochondrial survival signalling via mitochondrial ATP-sensitive K^+^ channel (mK_ATP_), while at the same time integrating feedback signalling from the mitochondria. Thus, given the large number of PKCε dependent pathways, this leaves much room for pinpointing the mechanism of quercetin in protection against IR injury. As an example of integrating cytoplasmic signalling and feedback signalling from mitochondria and intracellular stores, uncoupling increases the cytoplasmic [Ca^++^] [13,14], and transient increases in cytoplasmic [Ca^++^] can cause a strong activation of PKC, leading to preconditioning [15]. Previously, quercetin has been shown to uncouple mitochondria and, depending on experimental model, reduce the levels of oxidative stress [16,17]. As such, interaction between quercetin induced uncoupling and the PKCε pathway is a further putative mechanism for quercetin's cardioprotective effect.

The cardioprotective mechanism of DHS is also far from clear cut, with different strands of evidence pointing to its role as a release agent, uncoupler and PKCε pathway activator [17,18,8]. However, it remains unclear to what extent these mechanisms may be connected.

It is well known that, in contrast to certain pharmaceutics with a single, clear cut effect, polyphenols are notoriously promiscuous, having numerous biological targets and, as a result, a spectrum of mechanisms and effects which can be difficult to disentangle. With this in mind, we conducted an investigation into the uncoupling effect of quercetin and DHS in cell culture. Our aim was to gain an understanding of whether changes in energetic status, as well as mitochondrial and cytoplasmic [Ca^++^] upon treatment with these compounds are consistent with what one would expect if their protective effects were due to their uncoupling properties.

Hypothesizing that cardioprotection by quercetin and DHS may originate at the mitochondrial level, we investigated the effect of quercetin and DHS on the respiration of whole cells following exposure to normoxic and hypoxic conditions by use of high resolution respirometry. We then used a digitonin permeabilised cell model to more closely investigate the effect of these compounds on respiratory chain complexes (mainly I and II), under both hypoxic and normoxic conditions. This model was used here, because unlike whole cells, it allows the use of individual respiratory substrates to investigate various components of the respiratory chain. Once the slight increase in proton leak respiration (as measured by state IV respiration with oligomycin) and protective effects on complex I had been established, we used fluorimetry and fluorescence microscopy to investigate whether the consequences of an increased proton leak (decreased mitochondrial membrane protential, decreased intramitochondrial and increased cytoplasmic [Ca^++^]) could be observed. Mitochondrial [Ca^++^] was probed both in normoxia and following hypoxia. On the other hand, cytoplasmic [Ca^++^] and plasma membrane potential were only probed under normoxia due to technical limitations of the probes and protocols used.

## Materials and methods

### Materials and reagents

Dulbecco's modified Eagle's medium 5976 (DMEM), heat-inactivated fetal bovine serum (FBS), stabilised penicillin-streptomycin (PenStrep), sterile dimethylsulphoxide (DMSO), sodium lactobionate, quercetin, digitonin, atractyloside, FCCP, and HEPES were obtained from Sigma-Aldrich (United States). 2,3-Dehydrosilybin was a gift from Professor Vladimír Křen (Czech Academy of Sciences) [19]. Inorganic salts for the preparation of buffer solutions were obtained from Lachner (Czech Republic). Lipofectamine, OptiMEM, Fluo4 and JC-1 were obtained from Invitrogen (United States). Plasmids were a gift provided through Addgene [20,21,22]. H9c2 cells were obtained from European Collection of Authenticated Cell Cultures (ECACC No. 88092904; rat DX1X heart myoblast).

### H9c2 cell culture

H9c2 cells were maintained in DMEM with 10% FBS, 1% non-essential amino acids (NEAA) and 50 μg/ml PenStrep (cultivation medium) at 37°C, in an atmosphere containing 5% CO_2_. Cells were passaged 1:3 at 80% confluence and used between 5th and 20th passages.

For imaging procedures and hypoxia experiments cells were incubated in HEPES buffered solutions (NaCl 120 mM, HEPES 20 mM, Glucose 15 mM, NaOH 10 mM, KCl 5 mM, CaCl_2_ 2.3 mM, MgCl_2_ 1.6 mM, pH 7.4) with or without glucose or Ca^++^. For oxygraphy experiments investigating respiratory complex activity and one mechanistic experiment measuring [Ca^++^], MiR05 medium was used.

### Transient transfection

For transfection, cells were passaged and plated on 96-well plates (Nunc or In Vitro Scientific) at 10^4^ cells per well and cultured for 24 h under standard conditions. Cells were then transfected with plasmid-Lipofectamine mixture by a modified standard procedure with 0.5 μg: 0.5 μl: 25 μl, DNA: lipofectamine: OptiMEM per well. After transfection cells were incubated for a further 48 h prior to use.

### Hypoxia treatments

Three different hypoxia protocols were used. In brief, cells were incubated in glucose-free HEPES buffer under a humidified atmosphere of N_2_ for either 3 hours (oxygraphy or fluorimetry) or for 1 hour (microscopy).

For oxygraphy H9c2 cells were cultured until they had reached 80% confluence. Cells were then subjected to hypoxia. Subsequently these cells were trypsinised and suspended in either DMEM (protocol A) or MiR05 (protocol B) at one T 175 flask per polarigraphic chamber.

Cells used in the fluorimetric study with JC-1 were plated on 96-well plates at 10^4^ cells per well, and following 48 h loaded with JC-1 as described. Cells were then subjected to hypoxia. After hypoxia, cells were incubated with treatments for 15 minutes in HEPES buffer. Thereafter, buffer was exchanged for fresh HEPES buffer and measurements performed.

For fluorescent microscopy of mitochondrial [Ca^++^] H9c2-CEPIA3mt stable cells were cultured on 20 mm plates for 48 hours. Cells were then subjected to hypoxia for 1 hour. Buffer was exchanged rapidly for HEPES buffer containing the appropriate treatment and the imaging procedure commenced immediately. Shorter incubation time for microscopy experiments was used to reduce number of visual fields made unusable by cell death and detachment.

### High resolution respirometry in whole or permeabilised cells

**Fig 1:**
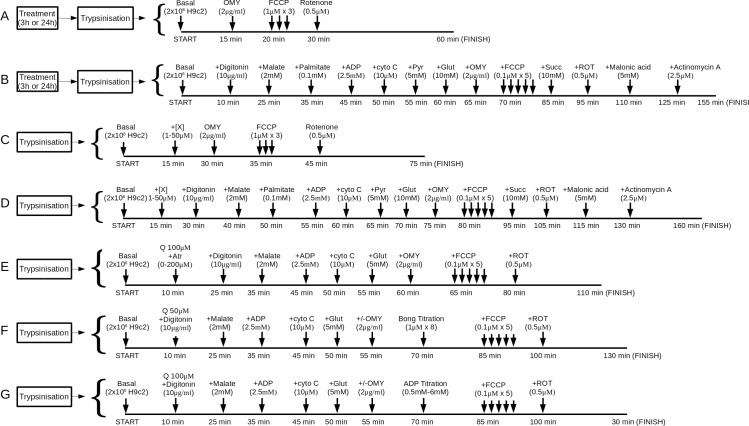
Oxygraphy protocols. This set of block diagrams details the measurements that were made in each of the seven different oxygraphy protocols used in this study. For each protocol the figure shows the final concentration of, and the duration of incubation with, each substrate, permeabilising agent and inhibitor. Prior to the start of each protocol, the polarigraphic chamber was equilibrated, and cell suspension prepared, with the appropriate medium (DMEM for protocols A and C, MiR05 for B, D, E, F and G). Each measurement was started after the cells had been injected into the polarigraphic chamber and allowed to equilibrate for 5–15 min. Protocols A and B were used to investigate the effects of hypoxia, or long term treatment with compounds of interest, on respiratory control ratios in whole cells (A) and respiratory complexes in permeabilised cells (B). Protocols C and D were used to investigate the effects of short term treatments with our compounds of interest on respiratory control ratios (C) and individual respiratory complexes (D). In these protocols “+[X]” indicates addition of quercetin or DHS. Protocols E, F and G were used to investigate mechanistic aspects of quercetin's effects, especially regarding ANT dependence and [ADP] dependence. For protocols C-G, cells did not undergo any treatment prior to trypsinisation and were treated with compounds of interest (or vehicle control) only *after* injection into the polarigraphic chamber. For protocol A, cells were either pretreated with compounds of interest (or vehicle control) for 24 h in culture (long term normoxia experiment) or subjected to 3 h of hypoxia, with or without treatments (whole cell hypoxia experiment), *prior to* trypsinisation and injection into the polarigraphic chambers. For protocol B, cells were likewise subjected to 3 h of hypoxia, with or without treatments, *prior to* trypsinisation. Protocol E was used to investigate the effect of atractyloside on quercetin's uncoupler-like effect. In each experiment, a single concentration of atractyloside (maximum of 200μM) was used. Protocols F, and G, where cells were titrated with either ADP (G) or bongkrekic acid (F), were carried out in two modes: i) to measure state III respiration, and ii) to measure oligomycin induced state IV respiration. Thus, oligomycin was only added to the chamber in these experiments when the protocols were set to measure state IV respiration. This was not applicable in protocol E, wherein a full set of inhibitor additions, and thus a full analysis, was performed for each concentration of atractyloside. Abbreviations used are as follows: “Atr”- atractyloside; “bong”- bongkrekic acid; “cyto c”- Cytochrome c; “Glut”- glutamine; “OMY”- oligomycin; “Pyr”- pyruvate; “Q”- quercetin; “ROT”- rotenone, “Succ”- succinate.

Respirometry was performed using an Oxygraph-2k (Oroboros, Austria). Before and after every experiment the polarigraphic chamber was cleaned by a standard protocol. Each chamber was pre-equilibrated with 2 ml of the appropriate medium for approximately 1 h at 37°C. Subsequently, excess medium was removed and 2×10^6^ cells suspended in approximately 300 μl of DMEM or MiR05 were added to each chamber. The experiment was then commenced in accordance with the appropriate protocol, as depicted in Fig 1. For the study of respiratory control ratios whole H9c2 cells in DMEM were used. For the study of respiratory complex activity, cells suspended in MiR05 were permeabilised with digitonin (final concentration 10μg/ml) and subsequently exhibited similar behaviour to isolated mitochondria.

### Probing mitochondrial potential with JC1

H9c2 cells were seeded on 96 well fluorescence plates (Nunc) at 10^4^ cells per well. At 48 h after plating, cells were incubated with 10 μM JC-1 in serum-free DMEM (SFM) for 20 min. Medium was then changed to fresh SFM and cells were subjected to hypoxia or normoxia, followed by 15 min treatment with test compounds. Medium was then changed to HEPES buffer and fluorescence measured using a Tecan Magellan 200M (Tecan, Switzerland) with Ex/Em_1_/Em_2_ of 485 nm/525 nm/590 nm, respectively.

### Production of CEPIA & GECO stable cell lines

In brief, H9c2 cells were trypsinised and transfected with pCMV-CEPIA3mt or CMV-mito-R-GECO1 plasmid (16 μg per 10^6^ cells) while suspended in OptiMEM at 2×10^5^ cells per ml. Cells were then plated on 100 mm culture dishes (Nunc) at 2×10^6^ cells per plate. Cells were maintained in selection medium until resistant colonies emerged (cultivation medium + 1 mg/ml G418). Selection medium was changed every 48 h. These were visually inspected under fluorescent light and colonies containing a high proportion of fluorescent cells sub-cloned. Of these, several were expanded for further use and stocks frozen (90% FCS, 10% DMSO) in liquid nitrogen. Stable cell lines were maintained in cultivation medium supplemented with 500 μg/ml G418.

### Microscopy and image processing

Zeiss spinning disk confocal microscope (Axio Observer Z1, ×40 objective) was used for time courses of membrane potential (Arclight) and intracellular [Ca^++^] in H9c2 cells. For excitation, Yokogawa fibre was used at 488 nm, with emission collected at 506 nm.

Zeiss AxiovertC epifluorescent microscope (×40 objective) with a Zeiss AxioCam ICM1 was used for time courses of intramitochondrial [Ca^++^] (CEPIA and GECO) and membrane potential (Arclight or DIBAC_4_(3)) with excitation by a HBO50 mercury lamp with a 470 nm/515 nm Ex/Em filter block for green and a 546 nm/608 nm Ex/Em block for red fluorescence. Intensity was measured in ImageJ following background subtraction. For each measured field, one well, or one 20mm dish, was used. Each field was chosen at random. Visual fields measured 156μm×156μm (confocal) or 460μm×345μm (widefield). For each repeat, 2–5 fields were processed and the average normalised intensity measured (ImageJ). Data are presented as the mean and standard error of the mean (SEM) of at least 4 repeats.

### Probing cellular [Ca^++^] with Fluo4

H9c2 cells were seeded on 96-well high performance optical plates. Cells were probed 48 h *post* plating with 2 μM Fluo4 by a modified standard procedure. Measurements were made either in standard HEPES buffer, Ca^++^ free HEPES or MiR05 medium with nifedipine (see supplementary data). Cells were imaged by confocal microscopy over 15 min at 0.5 frames per second (fps) with fluorophore excitation at 488nm by Yokogawa fibre (20% fibre output, 300 ms exposure, 1700 ms rest). An initial period of equilibration (60-270s depending on experiment) was followed by gentle addition of treatment (×2) in an equal volume of buffer. In later experiments frame rate was reduced to 0.25 fps in order to reduce data intensiveness.

### Probing mitochondrial [Ca^++^] with GECO or CEPIA

For normoxia experiments, H9c2 cells stably expressing mGECO1 were seeded on 96-well plates. Medium was changed 24 h *post* plating to 100 μl HEPES buffer per well. Following an initial period of recording, 100 μl of buffer with treatment (×2) was gently added to the well and recording continued till the end of the experiment. For hypoxia experiments, H9c2 cells stably expressing CEPIA3mt were seeded in 20 mm dishes. Following 24 h, cells were subject to 1 h hypoxia. Medium was then changed swiftly to HEPES buffer with treatments and recording commenced immediately. For GECO, images were collected at 0.1 fps (1.4 s exposure, 8.6 s rest) for 10 min. For CEPIA post hypoxia, images were collected at 0.033 fps (1.4 s exposure, 28.6 s rest) for 30 min.

### Probing plasma membrane potential with “Arclight-Q239”

H9c2 cells were seeded on 96-well plates or high performance optical 96-well plates and cultured for 24 h. Subsequently cells were transfected as detailed above. Medium was exchanged for HEPES buffer 48 h *post* transfection. Recording was carried out for 10 min at 1 fps for fluorescent microscopy or 20 min at 0.67 fps with excitation at 40% output, 500 ms exposure, 1000 ms rest, for confocal microscopy. Following an initial period of recording, treatment (×2) was added gently in an equal volume of HEPES and recording continued.

### Statistics and other information

Statistical analysis of data was performed using R (R Core Team, Austria). Analysis of Variance (ANOVA) with Tukey's *post*-*hoc* test, or T-test with Sidak correction (as appropriate) were the primary methods of assessing significance (P < 0.05 was considered statistically significant). Further details pertaining to methods used in this study can be found in the supplementary data (S1 File).

## Results

### Respirometry study in whole and digitonin permeabilised H9c2 cells

**Fig 2:**
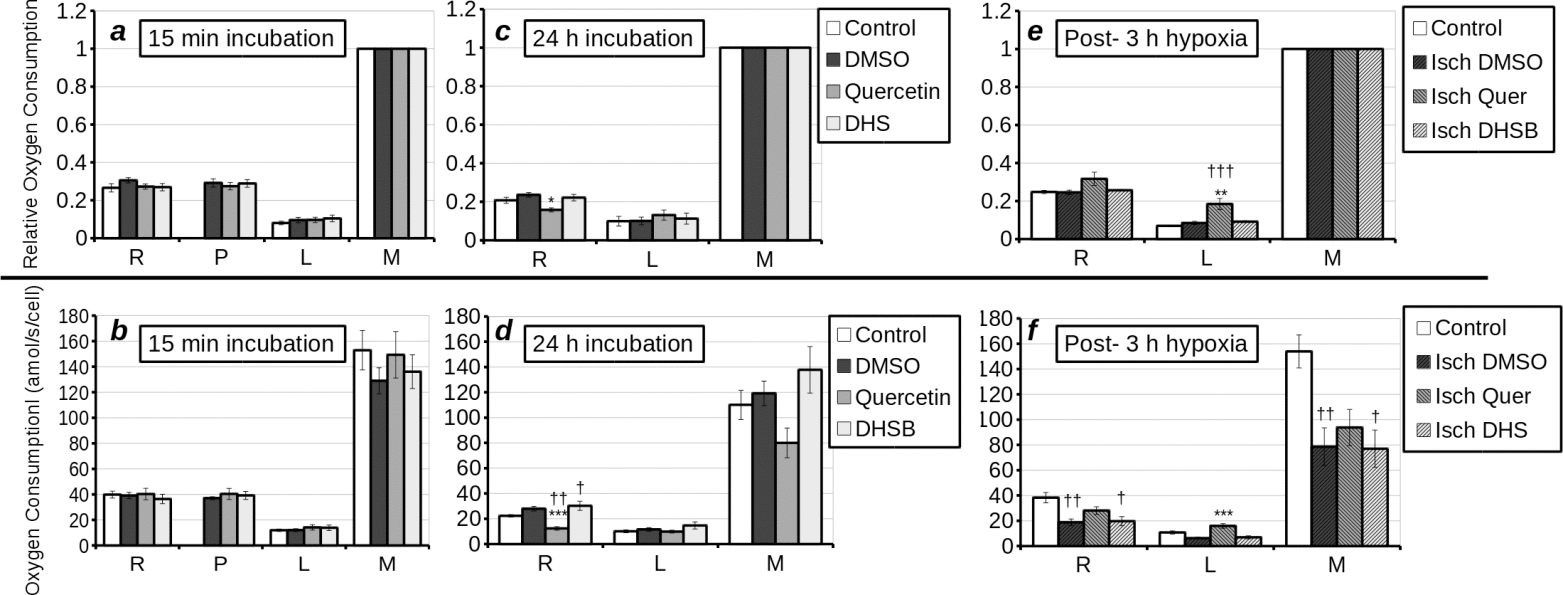
Respiratory parameters of whole **(a-f)** H9c2 cells treated with nothing (white bars), DMSO (dark grey bars) quercetin (25 μM) (grey bars) or DHS (1 μM) (light grey bars) as measured by high resolution respirometry. With respiratory control ratio (oxygen consumption relative to maximum oxygen consumption) in **(a, c, e)** and absolute rate in **(b, d, f)**. **(a, b)** Cells were treated with compounds for 15 min (protocol A). **(c, d)** Cells were treated for 24 h with compounds prior to oxygraphy (protocol B). **(e, f)** Cells were subject to 3 h of hypoxia (with the exception of “control”- which consisted of untreated, normoxic control cells) followed by oxygraphy with treatment carried out during the 3 h of hypoxia (Protocol B). Processed respiratory parameters derived from measurements of routine (R), treated (P), leak (L), maximum (M) adjusted for residual respiration are displayed on the x-axis. It should be noted that “R” represents resting coupled state III respiration, “L” represents state IV respiration (treatment + oligomycin), and “M” represents uncoupled respiration (L+FCCP). Statistical significant differences from vehicle-control were denoted by * (p < 0.05), ** (p < 0.01) and *** (p < 0.001) and from untreated control by † (p < 0.05), †† (p < 0.01) and ††† (p < 0.001).

As previous results have shown that at concentrations above 1 μM DHS has potentially damaging inotropic effects on the rat heart [15], in this experiment we used 1 μM DHS. High resolution respirometry revealed that 15 min incubation with 25 μM quercetin, or 1 μM DHS did not alter respiratory parameters in whole H9c2 cells (Fig 2A and 2B). However, 24 h incubation with 25 μM quercetin reduced both relative (P < 0.05) and absolute (P < 0.01) basal cell respiration (routine) rate (Fig 2C and 2D). Absolute uncoupled respiration rate was also reduced, but the change was not statistically significant. It is noteworthy that, following 24 h incubation, 1 μM DHS slightly increased routine respiration relative to untreated control but not when compared to 0.1% DMSO. Following 3 h hypoxia, the absolute resting and maximum respiratory rate (state III coupled respiration and uncoupled respiration respectively) are significantly reduced in hypoxic DMSO control cells (P < 0.01) and DHS treated cells (P < 0.05), but the reduction in quercetin treated cells is not statistically significant (Fig 2E and 2F). Quercetin significantly increased relative (P < 0.01) and absolute (P < 0.001) leak (state IV) respiration in H9c2 cells that had undergone 3 h hypoxia, when compared to hypoxia control cells. The relative leak respiration rate with quercetin was also higher than in normoxic control cells (P < 0.001). Thus we can deduce that long term treatment with quercetin reduced the rate of respiration in whole H9c2 cells, but increased the leak respiration in damaged H9c2 cells. DHS stimulates respiration in H9c2 cells if given sufficient time to enter the cells prior to starting the assay, but does not affect injured or healthy cells following 3 h of incubation.

**Fig 3:**
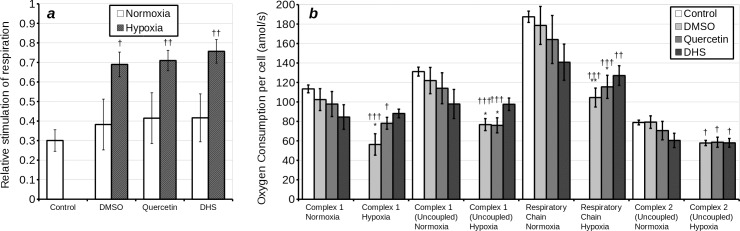
Respiratory parameters of permeabilised H9c2 cells treated with nothing (white bars), DMSO (dark grey bars), quercetin (25 μM) (grey bars) or DHS (1 μM) (light grey bars) as measured by high resolution respirometry (protocols C & D). Cells were incubated under normoxia (plain bars) or hypoxia (hatched bars) for 3 h, followed by oxygraphy with digitonin for permeabilisation. **(a)** Shows the differences in relative rate of respiration on malate + palmitate + ADP (relative to maximum respiratory rate of control permeabilised cells) prior to and following addition of cytochrome c, hence acting as a proxy for cytochrome c loss from mitochondria. **(b)** Shows the respiration of cells treated with DMSO, quercetin or DHS (following normoxia or hypoxia) on various substrates: Complex I respiration; “Complex I” (malate + palmitate + ADP + cytochrome c + pyruvate + glutamate), uncoupled respiration under complex I; “Complex I uncoupled” (“Complex I” + oligomycin +FCCP), uncoupled respiration under complex I and complex II; “Respiratory Chain” (“Complex I uncoupled” + Succ) and uncoupled complex II respiration; “Complex II uncoupled” (“Respiratory Chain” + Rotenone). Statistically significant differences from vehicle-control were denoted by * (p < 0.05), ** (p < 0.01) and *** (p < 0.001) and from untreated control by † (p < 0.05), †† (p < 0.01) and ††† (p < 0.001).

Hypoxia greatly increased the release of cytochrome c regardless of treatment (P < 0.01), as given by the proxy measurements of oxygen consumption by permeabilised cells prior to, and *post*, addition of cytochrome c (Fig 3A). Quercetin and DHS did not influence the loss of respiratory activity prior to addition of cytochrome c in cells treated for 3 h under normoxia or hypoxia (Fig 3A). 3 h of hypoxia caused a significant loss of complex I activity in vehicle treated cells when compared to normoxia treated (P < 0.05) and untreated (P < 0.001) controls (Fig 3B). Quercetin and DHS treatment partially recovered the loss of complex I activity relative to normoxic vehicle control (NS). However, there was no difference between uncoupled respiration at the level of complex I in quercetin and vehicle treated cells, unlike DHS treated cells, where partial recovery was observed. Full respiratory chain activity was reduced in hypoxia + quercetin (P < 0.05) and vehicle (P < 0.01) treated cells, with a partial (NS) loss of activity in DHS treated cells, relative to normoxic vehicle controls. Hypoxia treatment caused a statistically insignificant loss of complex II activity in H9c2 cells, which was not affected by treatment with either quercetin or DHS. From this, we deduce that quercetin does not protect the respiratory chain from damage during hypoxia under given experimental conditions. However, DHS may protect complex I upon reoxygenation. It is noteworthy that in the experiment using permeabilised cells, a model which is used for examining respiratory complex activity in a regulated environment and is considered to be an intermediate between whole cells and isolated mitochondria, DHS gave a visible effect, while in whole cells it did not. As DHS is very hydrophobic, the observation is perhaps due to the presence of digitonin in the second experiment allowing this flavonolignan to bypass the plasma membrane and access the mitochondria. Similarly, two other polyphenols (taxifolin and silybin) were tested at a final concentration of 25 μM, but there was no effect seen on respiratory parameters of H9c2 cells (p>0.05).

Taken together, these results show that the uncoupling effect of quercetin and DHS seen in isolated mitochondria is not directly translated to whole cells. Furthermore, this effect was only observed following prolonged incubation or in cells that had undergone hypoxia. Both compounds increased complex I respiration in *post*-hypoxic cells, but only DHS protected total complex I activity.

### Mitochondrial membrane potential

**Fig 4:**
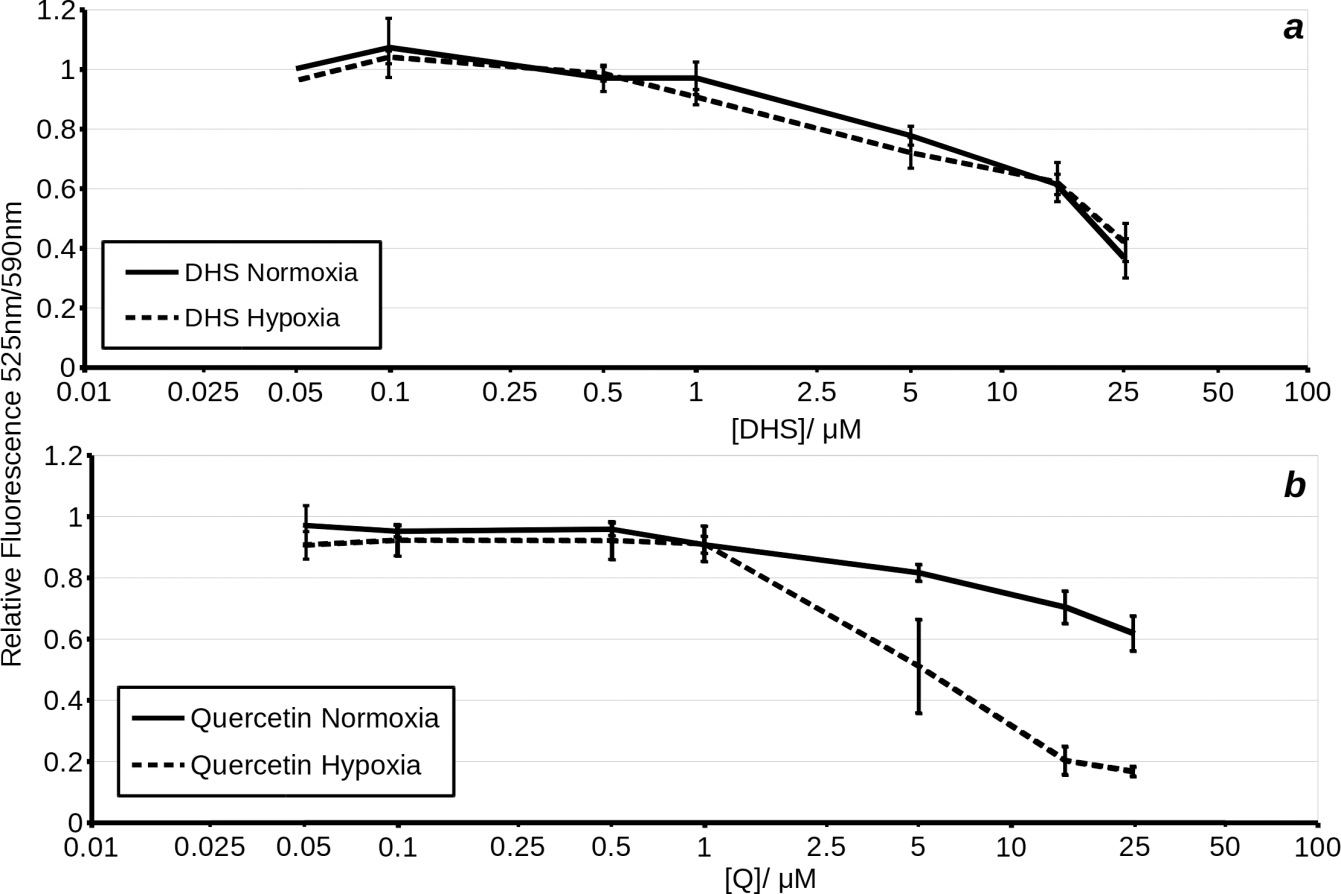
Loss of mitochondrial membrane potential, as indicated by ratiometric fluorimetry with JC-1 following treatment with DHS **(a)** and quercetin **(b)**. Cells were incubated with JC-1 for 1 h under hypoxia or normoxia, and subsequently measurements taken immediately. The solid line represents normoxia treated cells. The dashed line represents hypoxia treated cells.

As the results from the respirometry did not give a clear cut uncoupler effect in whole cells, we sought to clarify these observations by visualising mitochondrial membrane potential in H9c2 cells. Cells were loaded with JC-1, a semi-quantitative, ratiometric probe of mitochondrial membrane potential. Subsequently, after 3 h incubation under normoxia, they were incubated for 15 min with various concentrations of DHS or quercetin. Following this treatment, cells exhibited only a minor reduction in mitochondrial membrane potential at the higher concentrations of treatment (up to 25% reduction of Em_525nm_/Em_590nm_ with 25 μM quercetin) (Fig 4). Following 3 h under hypoxia, 15 min treatment (designed to mimic *post*-conditioning) with quercetin, but not DHS, caused a much stronger reduction of membrane potential (50% reduction of Em_525nm_/Em_590nm_ with 5 μM quercetin, 80% reduction with 25 μM quercetin).

### Adenine nucleotide transporter (ANT) dependence of quercetin uncoupling, in permeabilised H9c2 cells

**Fig 5:**
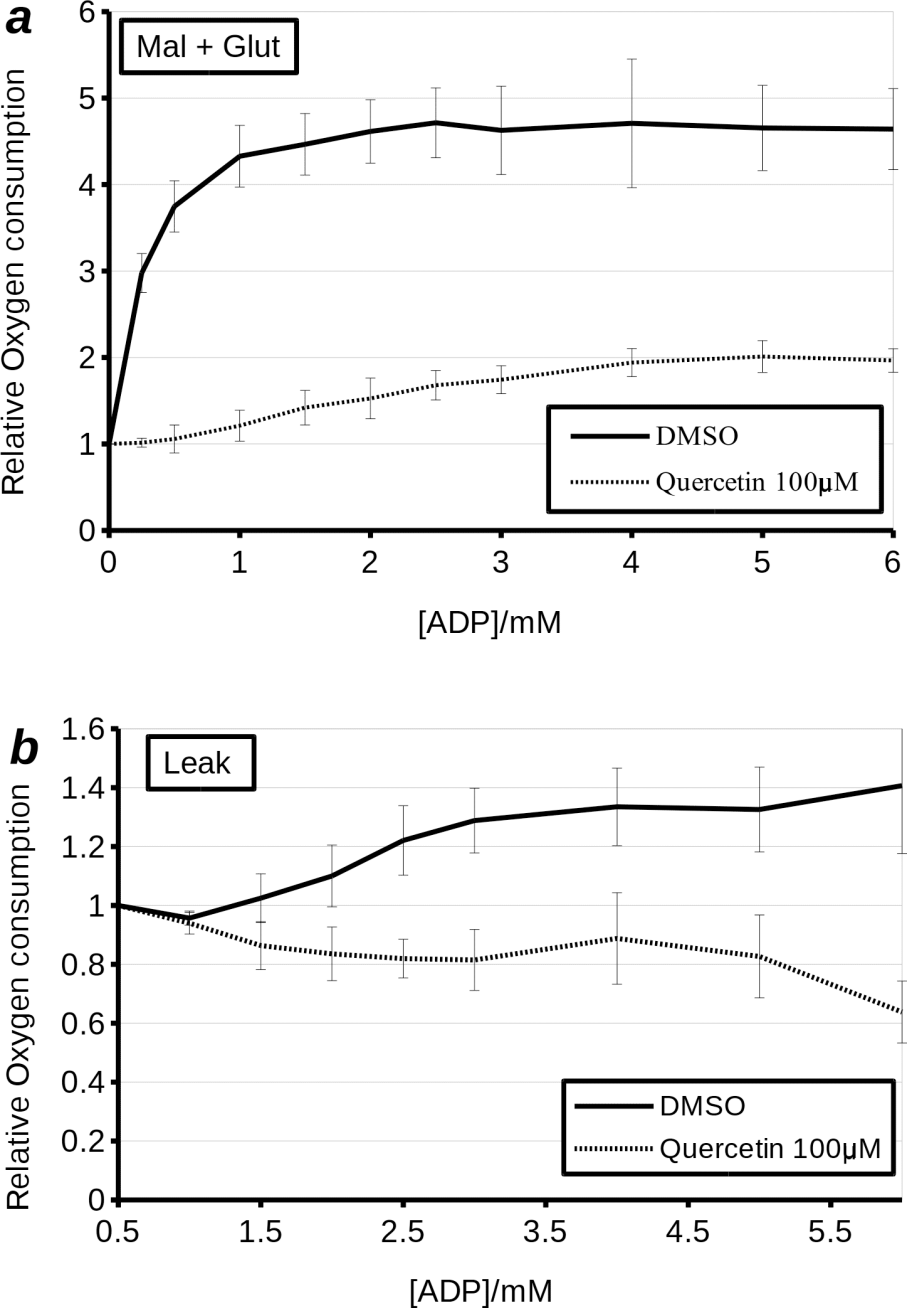
The effect of 100 μM quercetin on complex I (malate + glutamate)- i.e. state III coupled **(a)** and leak- i.e. state IV **(b)** respiration in permeabilised H9c2 cells treated with increasing concentrations of ADP. Quercetin appears to dramatically decrease both the V_max_ and the IC_50_ for stimulation of respiration by ADP in permeabilised H9c2 cells **(a)**. The solid black line represents cells titrated with increasing concentrations of ADP in the presence of DMSO. The dashed line represents cells treated with 100 μM quercetin and subsequently titrated with increasing concentrations of ADP.

**Fig 6:**
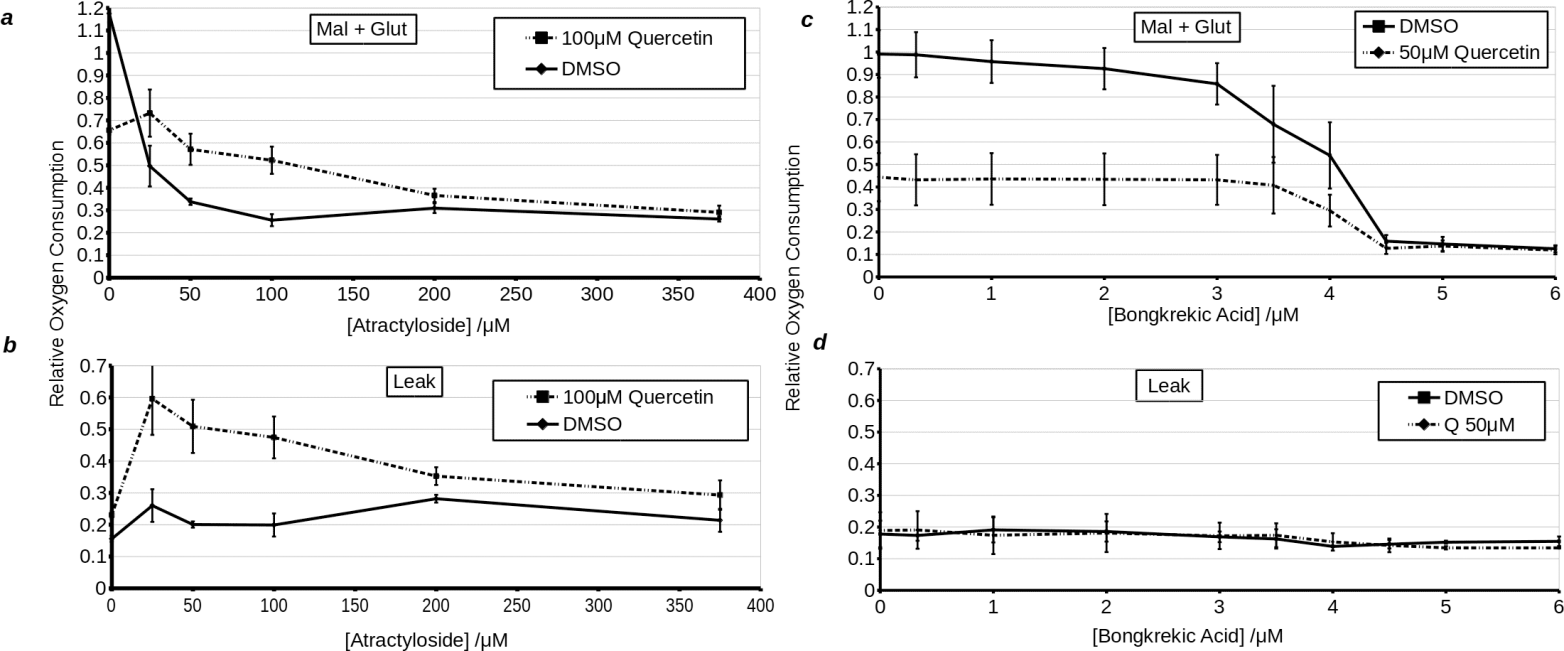
Effects of 100 μM quercetin on complex I (malate + glutamate)- i.e. state III coupled **(a, c)** and leak- i.e. state IV **(b, d)** respiration in permeabilised H9c2 cells treated with variable concentrations of atractyloside **(a, b)** or titrated with bongkrekic acid **(c, d)**. Solid line represents vehicle treated cells, while dashed line represents quercetin treated cells. *X*-axis shows the concentration of inhibitor in μM. *Y*-axis shows oxygen consumption relative to uncoupled respiration in DMSO treated cells.

Recently it has been shown that quercetin can transport H^+^ into liposomes by cycling through ANT [20] (Jaburek *et al*., private communication). Furthermore, quercetin has been shown to inhibit ANT, and it has been suggested that like atractyloside, it does so by locking ANT in the cytosolic facing configuration [11]. As this aligns with our experiments, which have shown that quercetin has a greater effect in cells having undergone hypoxia, we sought to perform additional experiments which would confirm ANT dependence of quercetin uncoupling. Firstly, we performed ADP titrations in digitonin permeabilised H9c2 cells treated with quercetin. The results showed that high concentrations of quercetin (100 μM) inhibited the enhancement of respiration by ADP (Fig 5). The IC_50_ was increased from less than 0.5 μM to approximately 2 μM. Likewise V_max_ was also decreased. Leak respiration, in line with total respiration, was stimulated with increasing [ADP] in vehicle treated cells, but was inhibited by increasing [ADP] in cells treated with quercetin. This indicates that quercetin interferes with an ADP dependent process and, conversely, that quercetin dependent uncoupler-like effect is reliant on molecular machinery that is affected by ADP. Atractyloside strongly inhibited oxygen consumption in permeabilised H9c2 cells respiring with substrates (state III coupled) and treated with vehicle or quercetin (Fig 6). Likewise, the use of high concentrations of quercetin (100 μM) inhibited mitochondrial respiration in permeabilised H9c2 cells. However, quercetin also blocked further inhibition of respiration by atractyloside, which was overcome at higher concentrations (200 μM) of the ANT inhibitor. Notably, as with high concentrations of quercetin (100 μM), atractyloside increased leak (state IV) oxygen consumption rate. Analysis of leak oxygen consumption rate revealed that quercetin does not *per se* reverse the effects of atractyloside. Instead, they act in a synergistic manner to increase leak oxygen consumption, an effect that is only counteracted at very high concentrations of atractyloside (above 200 μM). From this, we would draw the conclusion that the uncoupling effect of quercetin is not only ANT dependent, but moreover, is dependent on the inhibition of ANT. To further test this hypothesis, we repeated the experiment with bongkrekic acid, which inhibits ANT by locking it in a mitochondria-open conformation. Inhibition of respiration by bongkrekic acid was only slightly affected by quercetin. More interestingly there was no effect of bongkrekic acid on leak oxygen consumption in the presence of quercetin. This is also consistent with the conjecture, put forwards by Ortega and Garcia [16], that quercetin binds the c-state of ANT. This accords with our observations: When ANT is locked in the c-state by atractyloside, but not the m-state by bongkrekic acid, the uncoupler-like effects of quercetin were enhanced.

### Measurements of intramitochondrial [Ca^++^] by wide-field fluorescence microscopy

**Fig 7:**
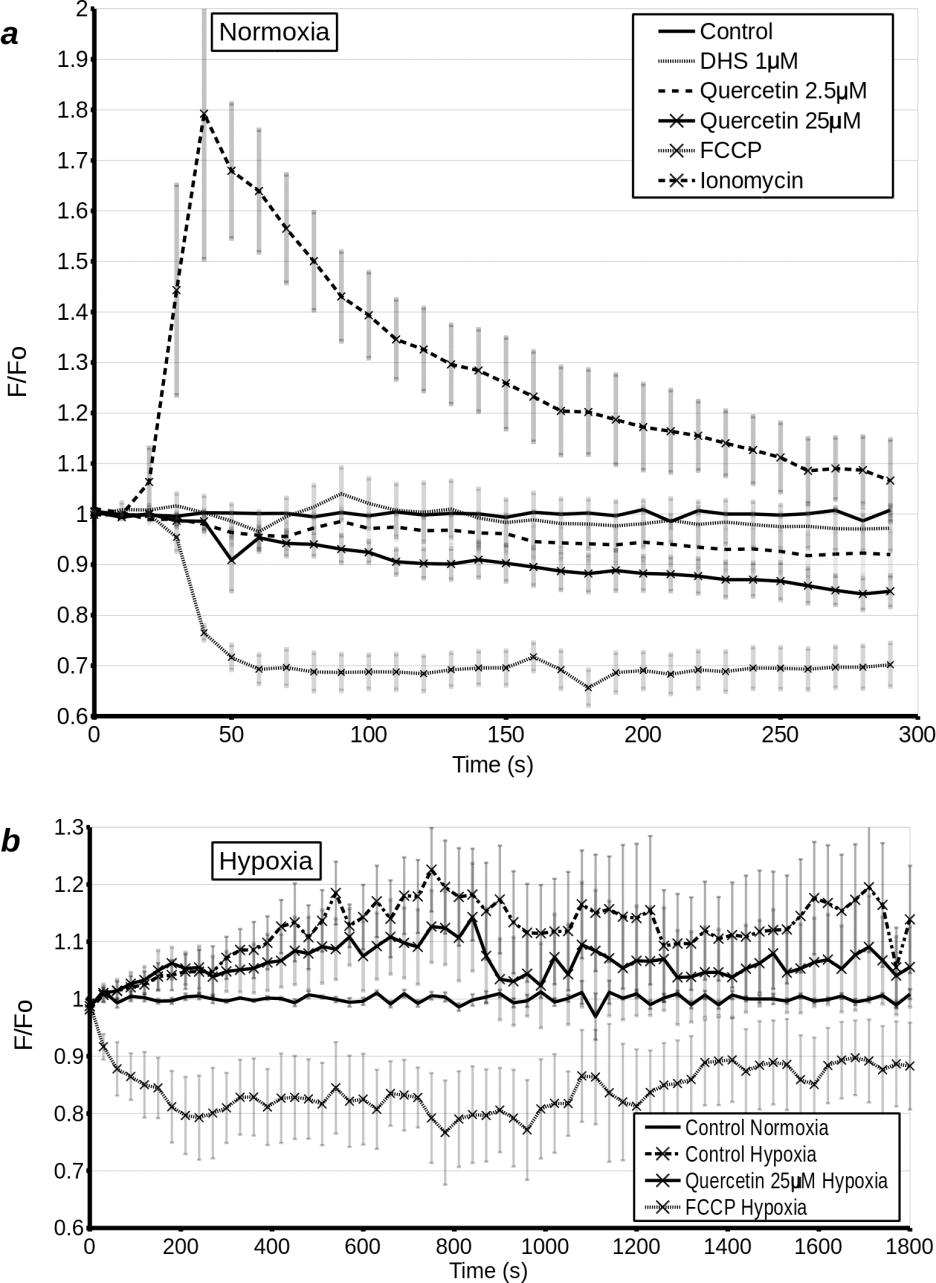
**(a)** Mitochondrial [Ca^++^] measured at 10 s intervals over 300 s by wide-field microscopy in H9c2 cells transfected with mGECO-1. Cells were treated with DMSO (control), 1 μM DHS (D1), 2.5 μM quercetin (Q 2.5), 25 μM quercetin (Q 25), 5 μM FCCP, or 1 μM ionomycin (Iono) 20 s after the start of the experiment. **(b)** Mitochondrial [Ca^++^] measured at *30 s intervals over 30 min (1800 s)* by wide-field microscopy in H9c2 cells transfected with mGECO-1, following 1 h normoxia or 1 h hypoxia. Cells were treated with DMSO (control normoxia/hypoxia), 25 μM quercetin or 5 μM FCCP immediately prior to the start of recording.

If, as the above results suggest, quercetin and DHS uncouple mitochondria in whole cells, we would expect a dissipation of the [Ca^++^] gradient on a similar time-scale to the dissipation of the [H^+^] gradient across the inner mitochondrial membrane. Ortega and Garcia [11] have previously shown a calcium efflux from mitochondria exposed to high concentrations of quercetin. To extend this finding to whole cells, we created a stable cell lines expressing [Ca^++^] dependent fluorescent proteins (rGECO-1 or CEPIA3) targeted to the mitochondria. Fluorescence microscopy showed that a large increase in cytoplasmic [Ca^++^], driven by ionomycin, also increased rGECO-1 fluorescence and hence mitochondrial [Ca^++^], while uncoupling of the mitochondria by 0.5 μM or 5 μM FCCP caused a rapid decrease in mitochondrial [Ca^++^] (Fig 7A). As expected from our respirometry data, at a concentration of 1 μM, DHS had no short term effect on mitochondrial [Ca^++^], while quercetin at 25 μM caused a small, gradual decrease in mitochondrial [Ca^++^]. It should be noted that 5 μM quercetin also caused an observable decrease in mitochondrial [Ca^++^], but the effect was not statistically significant and as such is questionable. We then went on to examine whether quercetin would have a similar effect on *post* hypoxic cells undergoing reoxygenation. H9c2 cells, stably expressing CEPIA, were subjected to 1 h hypoxia and immediately treated and imaged. There was a rapid increase in mitochondrial [Ca^++^] following hypoxia. This increase was attenuated by 25 μM quercetin and was completely reversed by 5 μM FCCP (Fig 7B). This experiment confirmed that the decrease in mitochondrial membrane potential caused by quercetin is accompanied by a decrease in mitochondrial [Ca^++^].

### Measurements of plasma membrane potential by wide-field fluorescence and confocal microscopy

**Fig 8:**
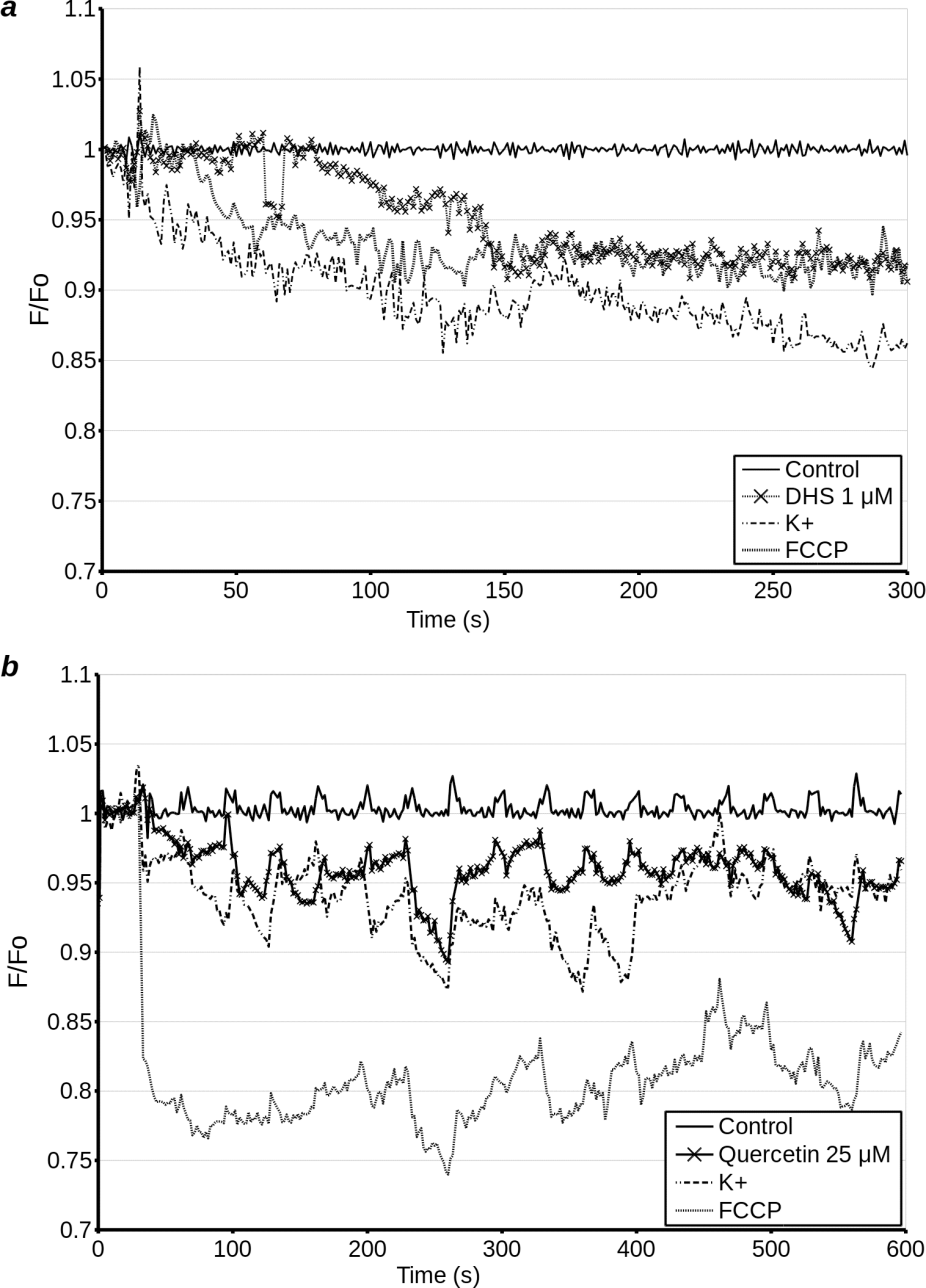
Plasma membrane potential in H9c2 cells transfected with Arclight potential sensor was measured by wide field microscopy over 300 s (treat 10 s after start of experiment). Cells were treated with 5 μM FCCP (FCCP), 1 μM DHS (DHS), 1 μM ionomycin (Ionomycin) or 140 mM K^+^ (K), 10 s into the experiment **(a)**. Confocal microscopy over 600 s (treatment 30 s after start of experiment) was used to establish the response of cells to treatment with quercetin. Cells were treated with 5 μM FCCP (FCCP), 15 μM quercetin (Q), 1 μM ionomycin (Ionomycin) or 140 mM K^+^ (K), 10 s into the experiment. **(b)**. Due to the crowded nature of this figure, error bars are omitted for clarity (see supplementary data, Fig A in S1 File).

Agents which de-energise the mitochondria are expected to lower intracellular ATP and hence eventually lead to the dissipation of plasma membrane potential. Furthermore, uncouplers such as FCCP are not completely specific, and at high concentrations will uncouple not only the mitochondria, but will also dissipate the plasma membrane potential directly. In addition, dissipation of plasma membrane potential prior to, or in the absence of, mitochondrial depolarisation can indicate a mechanism of action at the plasma membrane. Therefore, we tested the effects of quercetin, DHS and FCCP on plasma membrane potential by transfecting H9c2 cells with 'Arclight', a fusion protein consisting of modified pHluorin and phosphatase transmembrane domain, whose fluorescence increases with increasing membrane potential. All three compounds caused a decrease in F/F_0_ and hence plasma membrane potential (Fig 8). The decrease caused by FCCP was most marked and had the most rapid onset, with dissipation of plasma membrane potential and a drop in mitochondrial [Ca^++^] on a similar time scale. By around 400 s into the experiment, membrane potential of cells treated with FCCP was beginning to show a slight recovery. The effects of quercetin are difficult to compare. However, membrane depolarisation was both less pronounced and was still increasing towards the end of the experiment. It should be noted that despite not causing uncoupling or a decrease in mitochondrial [Ca^++^] on this time scale, DHS did cause a gradual decrease in plasma membrane potential, giving us further evidence that in the short term, the effects of DHS in whole cells may be due to interactions at the level of the plasma membrane.

### Measurement of cytoplasmic [Ca^++^] by confocal microscopy

**Fig 9:**
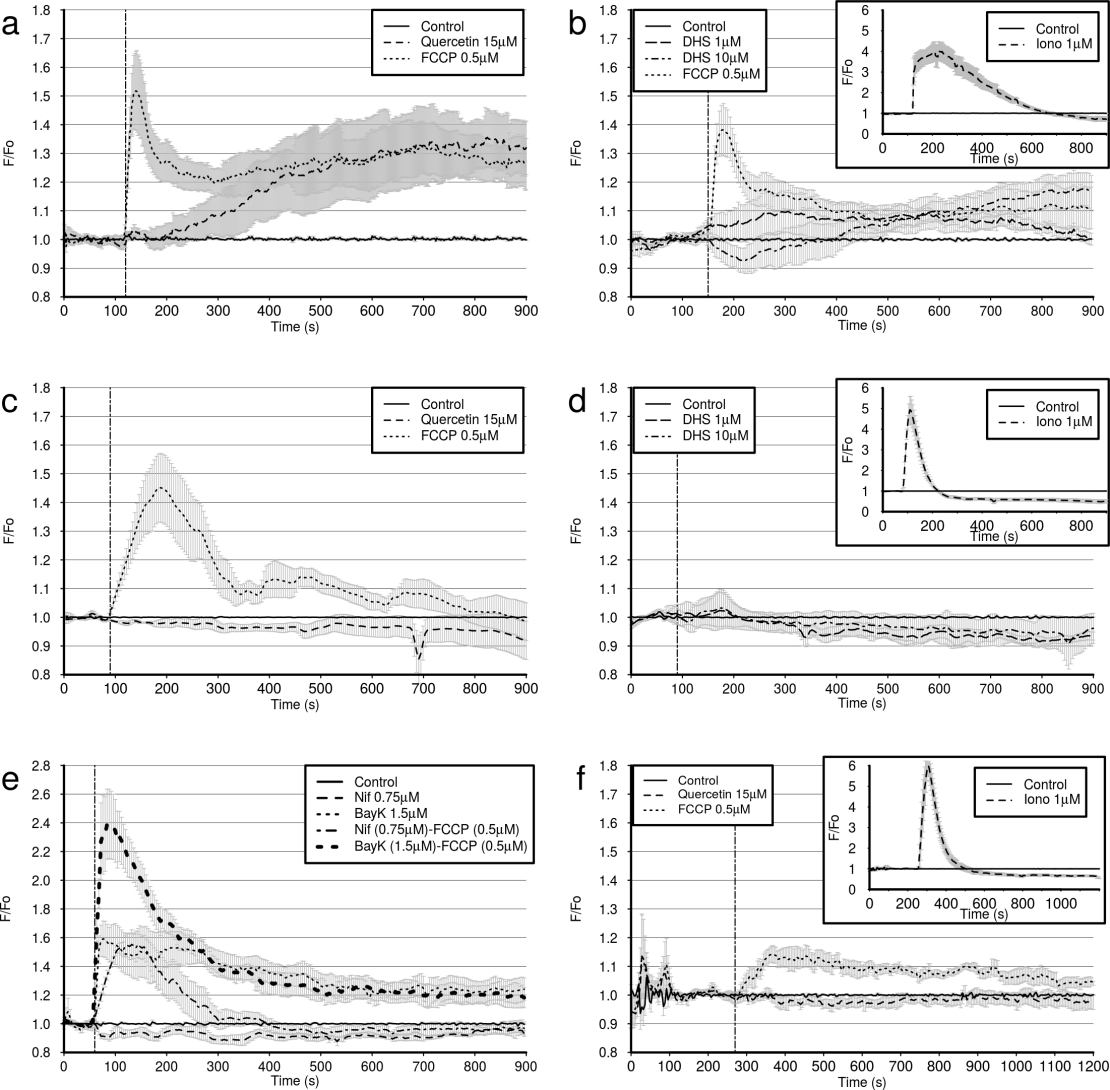
Confocal microscopy measurements of cytoplasmic [Ca^++^] (F/F_0_) in Fluo4 loaded H9c2 cells were used to further investigate the effects of quercetin, DHS and FCCP on Ca^++^ dynamics. After loading with 4 μM Fluo4-AM in HEPES, cells were unloaded in HEPES (**a, b, e,)**, HEPES with 0.75 μM nifedipine **(f)**, or Ca^++^ free HEPES **(c, d)**. Subsequently cells were imaged in standard HEPES **(a, b, e)**, calcium-free HEPES **(c, d)**, or calcium-free, sodium-free medium (MiR05) with nifepidine **(f)**. At the time point shown by the dashed vertical line, cells were treated with either DMSO (control) **(a-f)**, 15 μM quercetin **(a, c, f)**, 0.5 μM FCCP **(a, b, c, f)**, 1 μM or 10μM DHS **(b, d)** or 0.75 μM Nifedipine (Nif), 1.5 μM Bay K 8644 (BayK), Nif + 0.5 μM FCCP, BayK + 0.5 μM FCCP (**e)**. Cells were treated between 60s and 270s after the start of the experiment, as indicated by the dashed vertical line. Ionomycin (Iono), used as positive control, caused a rapid, and very strong increase in [Ca^++^] in all conditions **(insets- b, d, f)**, but in Ca^++^ free medium there was a rapid loss of Ca^++^ back into extracellular medium **(insets- d, f)**. Cells in MiR05 required a longer period of equilibration (270 s) **(f)**, than other groups. Positive control experiment with cells treated with 1 μM ionomycin against DMSO treated cells **(b, d, f; inset)**. Ionomycin causes a rapid influx of Ca^++^ into the cytosol.

Cytoplasmic [Ca^++^] is interdependent with mitochondrial [Ca^++^] and plasma membrane potential. Thus, a rapid release of mitochondrial or sarcoplasmic Ca^++^ can cause a corresponding increase in cytosolic [Ca^++^]. Plasma membrane depolarisation (including that caused by de-energising the cell) triggers Ca^++^ influx into the cytoplasm, which may or may not have a subsequent effect on intracellular store [Ca^++^], depending on various factors. A sufficiently large influx of Ca^++^ through the plasma membrane generally triggers Ca^++^ uptake to intracellular stores, mainly sarcoplasmic reticulum and mitochondria, followed by subsequent plasma membrane hyperpolarisation if a cell is not de-energised. Therefore, in order to further characterise the mechanism of action of DHS and quercetin on whole cells, we used confocal time-lapse microscopy to measure the cytoplasmic [Ca^++^] of H9c2 cells loaded with Fluo4-AM. Our results show that at 0.5 μM FCCP causes a biphasic response, with a rapid initial influx of Ca^++^ into the cytoplasm which peaks at approximately 50 s following addition, followed by a normalisation of [Ca^++^] and a slower secondary influx of Ca^++^ into the cytoplasm which peaks around 600 s *post* addition (Fig 9A and Fig 9B). This secondary response is not always observed in each field measured. The effect of 1 μM DHS, and 10 μM DHS was highly variable from cell to cell. However, on average, 1 μM DHS caused a gradual increase in fluorescence, followed by a return to baseline within 750 s of addition. 10 μM DHS, on average, caused a decline in intracellular [Ca^++^], which peaked approximately 50 s following addition, followed by a gradual increase in intracellular calcium (Fig 9A). Quercetin exhibited only the slow secondary response, which does not appear to peak until approximately 750 s *post* addition (Fig 9B). Ionomycin, used as a positive control, consistently causes an immediate, strong increase of intracellular fluorescence, followed by a gradual decay (Fig 9B, inset). It should be noted that due to the high variability of the cellular response in the measurements with DHS, these results should be interpreted with care.

Since there is still Ca^++^ present in the HEPES buffer, this experiment does not reveal whether the Ca^++^ which contributes to the increase in cytoplasmic [Ca^++^] comes from the emptying of intracellular stores. Therefore we repeated this experiment with calcium-free HEPES buffer. In this experiment neither polyphenol elevated [Ca^++^] (Fig 9C and 9D). Conversely, there may have been a slight decrease in [Ca^++^], but our methods were not sufficiently precise to confirm this. Curiously, under these conditions the initial peak [Ca^++^] with FCCP was delayed by approximately 1 min, and the secondary elevation in [Ca^++^] was not observed.

As the changes in cytoplasmic [Ca^++^] caused by the polyphenols appeared to be dependent on extracellular Ca^++^, we conducted an experiment to test whether the effects of DHS and quercetin on cytoplasmic [Ca^++^] resembled those of a classical opener (Bay K8644) and blocker (nifedipine) of Ca^++^ channels (Fig 9E). Our results show that Bay K8644 causes an immediate, sustained elevation in cytoplasmic [Ca^++^]. Nifedipine, on the other hand, had minimal effect on its own, but completely abrogated the secondary elevation of [Ca^++^] caused by FCCP, hence confirming that this secondary increase is dependent on extracellular Ca^++^. This experiment showed that the activity of the two polyphenols does not resemble that of direct openers or inhibitors of Ca^++^ channels.

Since we only observed a gradual release of Ca^++^ from mitochondria in quercetin treated cells, this still left the possibility that a loss of cytoplasmic Ca^++^ through calcium channel and NCX activity in calcium-free HEPES masks the rise in cytoplasmic [Ca^++^] from mitochondrial release. Therefore we repeated the experiment in a calcium and sodium free buffer in the presence of nifedipine. In this experiment quercetin did not cause an increase in cytoplasmic [Ca^++^] (Fig 9F). Curiously, under these conditions, the initial peak from FCCP is lost, but some elevation of [Ca^++^] is nevertheless observed. As the reverse mode of NCX is important in rapid depolarisation of the plasma membrane, and rapid calcium influx during action potential, this is understandable.

Thus, if we examine the effects of FCCP, a strong uncoupler, we observe that it causes a detectable increase in cytoplasmic [Ca^++^] purely by mitochondrial store emptying. At the same time, this signal is potentiated in the presence of extracellular sodium and calcium, presumably by the reversal of NCX and influx through Ca^++^ channels. Thus, FCCP causes both a direct increase in cytoplasmic [Ca^++^], and an indirect increase which is dependent on Ca^++^ channels. Quercetin, on the other hand, appears to raise intracellular [Ca^++^] in a manner that is dependent on the presence of extracellular Ca^++^, with no elevation of intracellular Ca^++^ detectable when NCX and Ca^++^ channels are not active. This could indicate that the elevation of cytoplasmic [Ca^++^] is not dependent on the loss of Ca^++^ from mitochondria caused by quercetin. Alternatively, we may be dealing with a form of calcium induced calcium release, but do not have sufficient sensitivity to detect the relatively small mitochondrial component of this increase in cytoplasmic [Ca^++^]. DHS, on the other hand, causes an increase in cytoplasmic [Ca^++^] which is dependent on extracellular Ca^++^, but previous experiments show no evidence of release of mitochondrial Ca^++^. The course of this activation is largely dissimilar to the increase in cytoplasmic [Ca^++^] caused by Bay K8644 induced channel opening, which suggests that we may be dealing with a signalling pathway-dependent increase in cytoplasmic [Ca^++^] as opposed to direct channel agonism.

## Discussion

This study reports effects of quercetin and DHS on respiratory status and [Ca^++^] gradients in H9c2 cells. The effects of the two compounds on mitochondrial polarisation, as well as mitochondrial and cytoplasmic [Ca^++^] were distinct. Both compounds caused some depolarisation of the plasma membrane, but stimulation of respiration was mild.

In permeabilised cells, uncoupling by quercetin was inhibited by increased [ADP] and enhanced by low concentrations of atractyloside, an ANT inhibitor binding the *c* state of ANT. Bongkrekic acid, an ANT inhibitor binding the *m* state of ANT, did not have this effect. This is consistent with previous observations in isolated mitochondria [16]. Similarly Jaburek *et al*. [23] have previously shown that ANT in a membrane is sufficient to enable quercetin mediated proton leak. Overall this evidence suggests that quercetin dependent uncoupling relies on *c* state binding to ANT. Alternatively it may depend on MPTP, which is favoured by the *c* state of ANT. This may explain the enhanced mitochondrial depolarisation by quercetin following prolonged hypoxia (Fig 4) and increased release of cytochrome c from isolated mitochondria following prolonged treatment with high concentrations of quercetin [16]. It should be noted that we did not observe the latter in our model with either quercetin or DHS. ANT inhibition by quercetin may well go some way in explaining its toxicity to cancer cells at high concentrations [16,24,25]. Furthermore, stimulation of respiration outside state IV may be masked by inhibition of either ANT or F_1_F_0_ ATPase [26,27].

Quercetin decreased mitochondrial [Ca^++^]. It also caused an increase in cytoplasmic [Ca^++^], but only in the presence of extracellular Ca^++^ (Fig 9). Since influx of extracellular Ca^++^ into the cytoplasm causes uptake of the ion by energised mitochondrial, these results indicate that the chain of events is unlikely to be initiated at the plasma membrane. Conversely, an influx of extracellular [Ca^++^] into the cytosol can be triggered by a modest release of Ca^++^ from intracellular stores. As transient increases in cytoplasmic [Ca^++^] can be cytoprotective, and studies have shown quercetin's protective effects to be dependent on PKCε and intracellular [Ca^++^] [2,28], it is plausible that an uncoupling induced released of store [Ca^++^] acts as a trigger for downstream events.

It should be noted that this study does not provide decisive evidence that uncoupling is the mechanism of quercetin induced cardioprotection. However, since uncoupling has been shown to be cardioprotective [11,12], the compounds' uncoupler-like properties are likely to be a contributing factor to their cardioprotective effects.

DHS has been shown to cause *post-*conditioning [7] as well as having an immediate, indirect inotropic effect on rat hearts [8]. Long term incubation of H9c2 cells with DHS seems to prevent some of the damage caused by hypoxia to the respiratory chain, but short term incubations at physiologically relevant concentrations have no immediate effect on respiratory parameters (Figs 2 and 3). Furthermore, DHS did not affect mitochondrial [Ca^++^], but did induce influx of extracellular [Ca^++^]. Taken together this suggests that on the level of tissues and organs, protection afforded by physiologically tolerated concentrations of DHS is unlikely to be caused by uncoupling. In this case pre- or *post-* conditioning is more likely to be mediated by potentiation of adrenergic signalling [8].

Our study has several quite self-evident limitations. Firstly, it should be noted that due to the high concentrations of polyphenols used (> 10 μM) our experiments do not fully reflect potential physiological situations, where concentrations of individual polyphenols would be unlikely to exceed 1 μM. Thus, this study does not answer the question of whether this mechanism would be important on the level of the tissues and organs. On the other hand, it has been shown that ANT mediated uncoupling can indeed lead to cardioprotection [29,30]. Thus, if submicromolar (and low micromolar) tissue concentrations of quercetin can cause ANT mediated uncoupling, then protective effects would likely be by this mechanism. Observations in whole hearts, however, suggest that at least for DHS, the initial inotropic effect precedes any mitochondrial effects in individual cells [18]. It is thus entirely possible that following initial action via cell surface receptors, these polyphenols then go on to affect the mitochondria. Alternatively, in the case of DHS, where some of the mitochondrial effects, notably depolarisation and [Ca^++^] gradient dissipation, are absent in whole cells, the changes in intracellular [Ca^++^] may be mediated entirely by crosstalk between survival pathways. It should be noted that changes in intracellular [Ca^++^] following treatment with DHS in cells probed with Fluo4 had an unusually high level of variability in comparison to the more consistent changes (or lack thereof) induced by other treatments. We are unable to fully explain this variability, but it is likely to be caused by one of three factors. Firstly, the measurements made with confocal microscopy use a small number of cells on a fairly small number of visual fields, therefore, it is not impossible, though unlikely, that this is due to statistical noise. Secondly, the stage in the cell cycle of any one measured cell is not generally known. Levels of both intracellular and cell surface receptors may vary with cell cycle. Thus if DHS exerts its effects through receptor binding at the cell surface, these effects may show more variability between cells than, for example direct ionophores such as ionomycin or strong uncouplers such as FCCP. The lack of significant changes in mitochondrial [Ca^++^] following DHS treatment, suggests that this may be the case. Next, polyphenols are known to be fickle, so it cannot be ruled out that the effects of DHS on cellular [Ca^++^] are dependent on initial intracellular [Ca^++^]. Since our experiments did not measure absolute, initial intracellular [Ca^++^], we are unable to confirm or refute this hypothesis. Lastly, DHS has a low solubility in aqueous solutions and the most variable group, the 10μM DHS treatment group, uses a near saturating concentration of DHS. Thus we may have observed threshold effects, with some cells having a much higher exposure to DHS than others. On the other hand, this is unlikely as microscopy is first and foremost a visual method, and we did not observe precipitate in this treatment group.

The second set of limitations relates to the fact that most of the methods used to measure cellular parameters in this study are fluorescence techniques. It is well known that investigators should be cautious about the choice of probe and platform, especially when working with compounds exhibiting autofluorescence or unspecific interactions. These can lead to meaningless results due to high background and quenching of probe, respectively. When probing cells with GFP-based genetically encoded fluorescence probes or Fluo4-AM, we have a fair degree of certainty that we have evaded the problems of quenching and changes in intracellular distribution of probe. Furthermore, by using microscopy instead of fluorimetry for most experiments, we are able to avoid, or compensate for, background fluorescence.

Thirdly, the methods we used have a low throughput. Therefore we only tested concentrations of quercetin and DHS that might be safe, though perhaps difficult to achieve by all but intravenous or intraperitoneal routes. The selection of these concentrations was based on available data from the literature and past experiments from our group, including Langendorf perfusions [18]. It is not clear, however, whether the concentrations achieved in perfused tissue or cell culture models are representative of unbound, and unconjugated, blood concentrations of these polyphenols. Since these compounds are hydrophobic, it is probable that they will be bound by albumins, and other serum proteins, lowering blood concentrations. This would attenuate the effects observed in perfusion models. In fact, studies have found that the binding constant of quercetin for albumin is in the order of 10^5^ M^-1^, meaning that at low micromolar concentrations around 80% of circulating quercetin will be bound [31, 32]. Conversely, as these compounds are somewhat hydrophobic, they have shown a propensity for bioaccumulation, which may lead to micromolar tissue concentrations [33] or low millimolar concentrations in cells over time [34]. As such, the results of any experiment that is not performed *in vivo* should be considered critically. It may be suggested that non-specific binding to cell components probably accounts for the differences observed between effects on respiration of whole cells reported here, and effects seen in isolated mitochondria. As the inotropic effects of DHS in rat heart were observed immediately upon perfusion, it is likely that bioaccumulation is not a major factor in this instance. However in experiments with a longer time scale (hours or days), this may become a factor for both quercetin and DHS.

Lastly, quercetin, when received by the oral route, is rapidly conjugated [35]. The biotransformations of DHS *in vivo* are not well studied, but as silybin is extensively conjugated [36], it is likely that DHS would undergo similar biotransformations. This is supported by *in vitro* findings showing glucoronidation and sulphation of DHS [37]. Quercetin from dietary sources is found in the unconjugated form, but more so in a myriad of conjugated forms, with studies generally reporting higher bioavailability of the conjugates [33]. As conjugates generally (but not always [38]) have a lower biological activity, we should expect that when quercetin or DHS are received by the oral route, they will be largely conjugated and the physiological activity will be lower than if they were to be received intravenously or intraperitoneally. As such the relevance of the results found here depends not only on the faithfulness of the model systems used in the study, but also on whether the compounds in question are viewed as dietary compounds or as potential therapeutic agents.

## Conclusion

In this study we investigated the protective effects of quercetin and DHS on mitochondria in H9c2 cells. We have found that in whole cells their uncoupler-like effects are attenuated, with subtle differences visible between DHS and quercetin. While they both partially protect the respiratory complexes of digitonin permeabilised cells *post* hypoxia, their mechanism of action and effects are distinct from what can be observed in physiological and sub-cellular systems. Most notably the effects of quercetin are generally consistent with a mitochondrial origin of the effect [39], namely mitochondrial uncoupling and reduced intramitochondrial [Ca^++^]. DHS, despite demonstrating a protective effect on respiratory parameters, does not appear to uncouple H9c2 cells (permeabilised or whole), or affect mitochondrial [Ca^++^]. On the other hand, given that it affected plasma membrane potential and whole cell [Ca^++^], we propose that in whole cells DHS has a primary site of action at the plasma membrane.

## Supporting information

S1 File Supplementary methods and information. Supplementary data file containing expanded methods as well as Figures A and B.
(PDF)

S2 File Open data archive. Open Data archive containing the minimal dataset behind all figures. Raw data can be made available upon request.
(ZIP)
