# Supplementary material for: On the causes and consequences of the uncoupler-like effects of quercetin and dehydrosilybin in H9c2 cells
Source: PLoS One. 2017 Oct 4;12(10):e0185691. doi: 10.1371/journal.pone.0185691 (PMC5627936; doi:10.1371/journal.pone.0185691)
Supplement: S1 File — Supplementary data file containing expanded methods as well as Figures A and B. (PDF) [file pone.0185691.s001.pdf]

On the causes and consequences of the uncoupler-like effects of quercetin and dehydrosilybin in H9c2 cells

Aleksey V. Zholobenko[1], Ange Mouithys-Mickalad[2], Zdenek Dostal[1], Didier Serteyn[2,3], Martin Modriansky[1]*

[1]Department of Medical Chemistry & Biochemistry, Faculty of Medicine and Dentistry, Palacky University, Olomouc, Czech Republic.

[2]Centre for Oxygen, R&D (CORD), Institut de Chimie, Sart-Tilman, Université de Liège, Liège, Belgium

[3]Faculté de Médecine Vétérinaire, Sart Tilman, Liège, Belgium.

*Corresponding author

E-mail: martin.modriansky@upol.cz (MM)

Extended Materials and Methods

Materials and Reagents

Dulbecco's modified Eagle's medium 5976 (DMEM), heat-inactivated fetal bovine serum (FBS), stabilised penicillin-streptomycin (PenStrep), sterile dimethylsulphoxide (DMSO), sodium lactobionate, quercetin, digitonin, atractyloside, FCCP, and HEPES were obtained from Sigma-Aldrich (United States). Dehydrosilybin was a gift from Professor Vladimir Kren at the Institute of Microbiology of the Czech Academy of Science [19]. Inorganic salts for the preparation of buffer solutions were obtained from Lachner (Czech Republic). Fluo4 and JC-1 were obtained from Invitrogen (United States). Bongkrekic acid was obtained from Enzo Life Sciences (United States). Arclight-Q239 (Addgene plasmid #36856) was a gift from V. Pieribone [20]. pCMV-CEPIA3mt (Addgene plasmid #58219) was a gift from M. Lino [21]. CMV-mito-R-GECO1 (Addgene plasmid #46021) was a gift from R. Campbell [22]. H9c2 cells were obtained from European Collection of Authenticated Cell Cultures (ECACC No. 88092904; rat DX1X heart myoblast).

H9c2 cell culture

H9c2 cells were maintained in DMEM with 10 % foetal bovine serum (FBS), 1 % non-essential amino acids (NEAA) and 50 µg/ml PenStrep (cultivation medium) at 37˚C, in an atmosphere containing 5 % CO_2. Cells were passaged at 1:3 when confluence reached 80 % and were used between 5th and 20th passages.

For imaging procedures and certain control experiments, HEPES buffer (NaCl 120 mM, HEPES 20 mM, Glucose 15 mM, NaOH 10 mM, KCl 5 mM, $CaCl_2$ 2.3 mM, $MgCl_2$ 1.6 mM, pH 7.4) was applied to cells. For hypoxic experiments glucose-free HEPES buffer was used (made up as HEPES buffer, but without glucose). For mechanistic experiments investigating the origins of treatment-induced Ca^{++} fluxes in H9c2 cells, Ca^{++} free HEPES buffer was used (made up as HEPES buffer, but without Ca^{++}, and with 5 mM EGTA). For oxygraphy experiments investigating respiratory complex activity and one mechanistic experiment measuring [Ca^{++}], MiR05 medium was used (Sucrose 110 mM, Potassium Lactobionate 60 mM, HEPES 20 mM, Taurine 20 mM, KH_2PO_4 10mM, $MgCl_2$ 3mM, EGTA 0.5mM, BSA 1 mg/ml). The application of these buffers and media is described in detail in the appropriate subsections below.

Transfection with "Arclight" plasmid

For transfection, cells were passaged and plated on flat bottomed 96-well plates (transparent (Nunc), or high performance optical plates (InVitro Scientific)) at 10^4 cells per well and allowed to grow for 24 h under standard conditions. Medium was then changed to OptiMEM (Life Technologies, United States) and cells incubated for 1 h. Cells were subsequently transfected with plasmid-lipofectamine (Life Technologies, United States) at 0.5 µg DNA and 0.5 µl lipofectamine in 25 µl OptiMEM per well and incubated for 4 h at 37°C, 5 % CO_2. Medium was changed to cultivation medium and cells were incubated for a further 48 h prior to use.

Treatment of Cells with DHS and quercetin

Quercetin and DHS stock solutions were prepared in DMSO, at concentrations of 25 mM and 10 mM respectively, and kept frozen at -20°C in single-use aliquots. For the treatment of cells, the compounds were generally diluted in treatment buffer/medium to a final concentration of 0.05 µM – 25 µM, as appropriate for each experiment. The exception is the use of a final concentration of 100 µM quercetin with oxygraphy protocol E-G (Fig. 1). All vehicle controls used a volume of DMSO which was identical to the volume of the treatment. The maximum concentration of DMSO in most experiments was 0.1% V/V, with the exception of oxygraphy protocols E-G, where it was 0.4%.

Hypoxia Treatments

This study used three regimes of hypoxia pretreatments, depending on whether cells were used for microscopy, fluorimetry or oxygraphy. In all three hypoxic regimes cells were incubated in glucose-free HEPES buffer under a humidified atmosphere of N_2. This type of hypoxic treatment is sometimes referred to as simulated ischemia or metabolic inhibition.

Cells to be used for oxygraphy (H9c2) were cultured in a T175 cell culture flask until they had reached a confluence of 80%. Growth medium was then exchanged for glucose-free HEPES buffer, supplemented with the appropriate treatment, and cells incubated at 37°C in a humidified atmosphere of nitrogen for 3 hours. Subsequently these cells were trypsinised and suspended in either DMEM (protocol A) or MiR05 (protocol B).

Cells used in the hypoxia branch of the fluorimetric study with JC-1 were plated on 96-well plates at 10^4 cells per well and following 48 h loaded with JC-1 as described. Cells were then incubated for 3 hours in glucose-free HEPES buffer at 37°C in humidified nitrogen atmosphere. At the end of this incubation, cells were then incubated with treatments for 15 minutes in HEPES buffer. Thereafter buffer was exchanged for fresh HEPES buffer and measurements carried out.

Cells that were to be used for fluorescent microscopy of mitochondrial [Ca^{++}] (H9c2-CEPIA3mt stable cells) were cultured on a 20 mm culture dish (2×10^5 cells per dish) for 48 hours. Subsequently medium was exchanged for glucose-free HEPES buffer, and cells incubated at 37°C in an atmosphere of nitrogen (humidified) for 1 hour. Buffer was then rapidly changed for 2 ml of HEPES buffer containing the appropriate treatment and the imagining procedure commenced immediately.

The reason for the different incubation times between the experiments was that the shorter incubation time for microscopy experiments was required to simplify the localisation and stability of cells in each visual field.

High Resolution Respirometry in whole or permeabilised cells

Respirometry was performed using an Oxygraph-2k (Oroboros, Austria). Before and after every experiment the polarigraphic chamber was cleaned by a standard protocol. Each chamber was pre-equilibrated with 2 ml of the appropriate medium for approximately 1 h at 37˚C. Subsequently, excess medium was removed and 2×10^6 cells (approximately one medium or one large flask, depending on experiment) suspended in approximately 300 µl of the appropriate medium were added to each chamber. The experiment was then commenced in accordance with the appropriate protocol.

Figure 1 shows the protocols used in the oxygraphy experiments. In protocols A and C (Fig 1), designed to examine respiratory control ratio in whole cells, the chamber was filled with DMEM. In protocols B, D, E, F & G (Fig 1) MiR05 medium was used.

Protocols E-G (Fig 1) were used for mechanistic studies of quercetin's possible interaction with ANT. These protocols tested how quercetin affects ANT inhibition by atractyloside (E) and bongkrekic acid (F), as well as how it affects the stimulation of respiration by ADP (G). The reason that Protocols (E) and (F) differ, is that while atractyloside is readily available, and a full analysis could be performed for each concentration, bongkrekic acid does not have such a high availability, and a serial titration was performed to make best use of limited stocks.

The parameters measured in protocols (A) and (C) were “Routine” or “R” (basal respiration of whole cells), “Treated” or “T” (basal respiration of whole cells following treatment with compounds of interest), “Leak” or “L” (state IV respiration of whole cells following addition of oligomycin) and “Maximum” or “M” (uncoupled respiration following addition of FCCP). All recorded values were adjusted for residual non-respiratory chain respiration, measured after addition of rotenone. It should be noted that “R” and “T” are the same in protocol (C). This is because cells were treated in culture *prior* to the experiment, and therefore the basal oxygen consumption measured is, by definition, oxygen consumption following treatment. Oxygen consumption was expressed either as an absolute value or as respiratory control ratio (absolute value divided by “M”). It should be noted that basal respiration of permeabilised cells in MiR05 is almost zero because respiratory substrates leak out and are diluted in the medium. Whole cells in DMEM, on the other hand, respire normally. Similarly, maximum (uncoupled) respiration in whole cells is generally not the same as maximum respiration in permeabilised cells. In the former, it is limited by physiological substrate concentrations, while in the latter the experimenter controls the substrate mixture, thus permitting the possibility of either a higher, or lower, maximum (uncoupled) rate of respiration depending on substrate mixture used.

Probing mitochondrial potential with JC1

H9c2 cells were seeded on black flat bottom 96 well plates (Nunc) at 10^4 cells per well. At 48 h after plating, cells were incubated with 10 µM JC-1 in serum-free DMEM (SFM) for 20 min. Medium was then changed to fresh SFM and cells were subjected to 3 h of hypoxia (N_2/CO_2 mix, 37˚C) or normoxia, followed by 15 min treatment with test compounds. Medium was then changed to HEPES buffer and ratiometric Fluorimetry was performed using a Tecan Magellan 200M (Tecan, Switzerland) with Ex/Em_1/Em_2 of 485 nm/525 nm/590 nm.

Production of CEPIA & GECO stable cell lines

We sought to investigate mitochondrial [Ca^{++}] using genetically encoded calcium indicators. In order to avoid having to transfect cells prior to stressful protocols (hypoxia followed by fluorescence microscopy), we sought to create a stable H9c2 based cell line expressing these proteins. In brief, H9c2 cells were trypsinised and transfected with pCMV-CEPIA3mt or CMV-mito-R-GECO1 plasmid (16 µg per 10^6 cells) while suspended in OptiMEM at 2×10^5 cells per ml. Cells were then plated on 100 mm cell culture plates (Nunc) at 2×10^6 cells per plate. 4 h *post*

plating medium was changed to cultivation medium. 24 h *post* plating cultivation medium was exchanged for selection medium (cultivation medium + 1 mg/ml G418). Selection medium was changed every 48 h. Cells were thus maintained until resistant colonies emerged. These were visually inspected under fluorescent light, with colonies containing a high proportion of fluorescent cells marked and sub-cloned into 96 well plates. Of these, several were expanded for further use and frozen stocks (90% FCS, 10% DMSO) preserved in liquid nitrogen. Stable cell lines were maintained in cultivation medium supplemented with 500 µg/ml G418.

Microscopy and Image Processing

With the exception of high background fluorescence measurements, where confocal microscopy was used to eliminate out of plane background emissions, widefield epifluorescence microscopy was used for construction of time courses. Zeiss spinning disk confocal microscope (Axio Observer Z1, ×40 objective) was used for visualising time courses of membrane potential (Arclight) and intracellular [Ca^{++}] in H9c2 cells. For excitation, Yokogawa fibre was used at 488 nm, with emission collected at 506 nm. Zeiss AxiovertC epifluorescent microscope (×40 objective) with a Zeiss AxioCam ICM1 was used for visualising time courses of intramitochondrial [Ca^{++}] (CEPIA and GECO) and membrane potential (Arclight or $DIBAC_4(3)$). Excitation was provided by a HBO50 mercury lamp in conjunction with a 470 nm/515 nm Ex/Em filter block for Arclight, and CEPIA and the 546 nm/608 nm Ex/Em filter block for GECO. Image intensity was measured in ImageJ following background subtraction. For each measurement, one well of a 96 well plate, or one 20mm dish, was used. Each field was chosen at random on the well/plate. For confocal microscopy a field measured 156µm×156µm. For widefield microscopy a field measured approximately 460µm×345µm. For each repeat, the average normalised intensity of several (2-5) fields (following background subtraction) was used, with photobleaching corrected to untreated control by a running average model. Data are presented as the mean and standard error of the mean (SEM) of at least 4 repeats.

Probing cellular [Ca^{++}] with Fluo4

H9c2 cells were seeded on 96-well high performance optical plates at 10^4 cells per well. Cells were then probed 48 h *post* plating by incubating in HEPES buffer with 2 µM Fluo4 (0.5 µl per ml of buffer with 0.5 µl/ml 50% pluritonic F-127) for 30 min at room temperature. Cells were unloaded by 30 min incubation in HEPES buffer, which was then aspirated and replaced with

fresh HEPES buffer. For measurements in Ca^{++} free HEPES, cells were loaded in standard HEPES buffer, but unloaded and measured in Ca^{++} free HEPES buffer. For measurements in MiR05, cells were loaded and unloaded in HEPES buffer, but the measurements themselves were made in MiR05 supplemented with nifedipine. MiR05 was used for mechanistic experiments when a sodium-free, calcium-free medium was required to test whether Ca^{++} release from mitochondria could be detectable when most movement of Ca^{++} across the plasma membrane had been arrested. In this final experiment a longer initial measuring time (270s) was required prior to treatment, in order to allow equilibration of intracellular [Ca^{++}]. Cells were imaged by confocal microscopy over 15 min at 0.5 frames per second (fps) with fluorophore excitation at 488nm by Yokogawa fibre (20 % fibre output, 300 ms exposure, 1700 ms rest). An initial period of equilibration was followed by gentle addition of treatment (×2) in an equal volume of buffer. In later experiments (extracellular Ca^{++} free experiments & channel agonist/antagonist experiments) frame rate was reduced to 0.25 fps in order to reduce data intensiveness.

Probing mitochondrial [Ca^{++}] with GECO or CEPIA

For normoxia experiments, H9c2 cells stably expressing CEPIA3mt or mGECO1 were seeded on transparent, flat bottomed 96-well plates at 10^4 cells per well. Medium was changed 24 h *post* plating to 100 µl HEPES buffer per well. Following an initial period of recording, an equal volume of HEPES buffer with treatment (×2) was gently added to the well and recording continued till the end of the experiment. For hypoxia experiments, H9c2 cells were seeded in 20 mm dishes at 2×10^5 cells per plate. Following 24 h, the medium was changed to glucose free HEPES buffer and cells incubated under hypoxia for 1 h. Following this, medium was changed to HEPES buffer with treatments and time lapse microscopy recording commenced immediately. For GECO, images were collected at 0.1 fps (1.4 s exposure, 8.6 s rest) for 10 min. For CEPIA post hypoxia, images were collected at 0.033 fps (1.4 s exposure, 28.6 s rest) for 30 min.

Probing plasma membrane potential with “Arclight-Q239”

H9c2 cells were seeded on transparent 96-well plates or high performance optical 96-well plates at 10^4 cells per well and cultured for 24 h. Subsequently cells were transfected as detailed above. Medium was exchanged for HEPES buffer 48 h *post* transfection (100 µl per well for fluorescent and 150 µl per well for confocal microscopy). For fluorescence microscopy, time lapse recording was carried out for 10 min at 1 fps. For confocal microscopy, time lapse recording was carried out

for 20 min at 0.67 fps, with fluorophore excitation by Yokogawa fibre (40 % output, 500 ms exposure, 1000 ms rest). Following an initial period of recording, treatment (×2) was added gently in an equal volume of HEPES and recording continued.

Statistics

Statistical analysis of data was performed using R (R Core Team, Austria). Analysis of Variance (ANOVA) with Tukey's *post-hoc* test, or T-test with Sidak correction (as appropriate) were the primary methods of assessing significance ($P < 0.05$ was considered statistically significant).

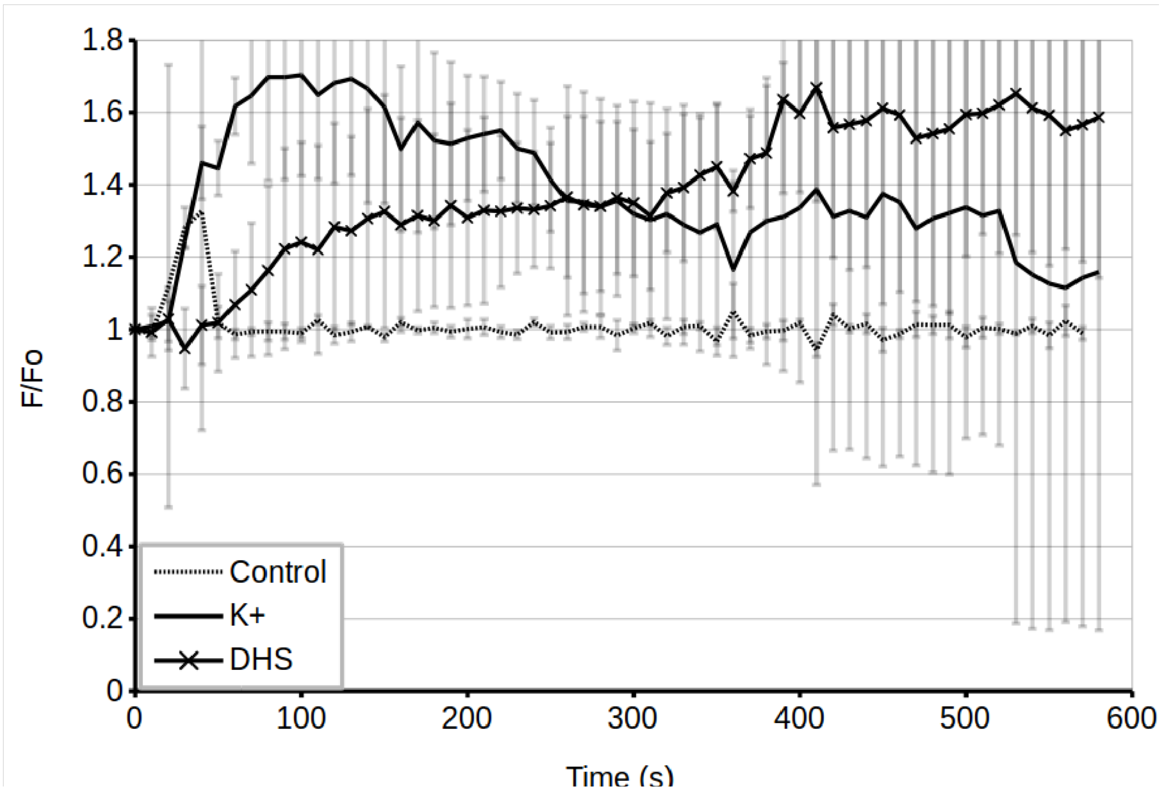

Fig A. Preliminary fluorescence microscopy with DiBAC$_4$(3). Prior to investigation of the effect of quercetin and DHSB on plasma membrane potential, we performed preliminary experiments, probing H9c2 cells with DiBAC. The fluorescence of this probe increases upon depolarisation of the plasma membrane. Cells were imaged using widefield epifluorescence microscopy, either with vehicle conditions (Control), with 140 mM K^+ in the buffer (K^+) or with 10 µM DHSB (DHS). An increase in fluorescence, indicating membrane depolarisation is observed. This also confirms that our observations with the Arclight construct for DHS are not an artefact.

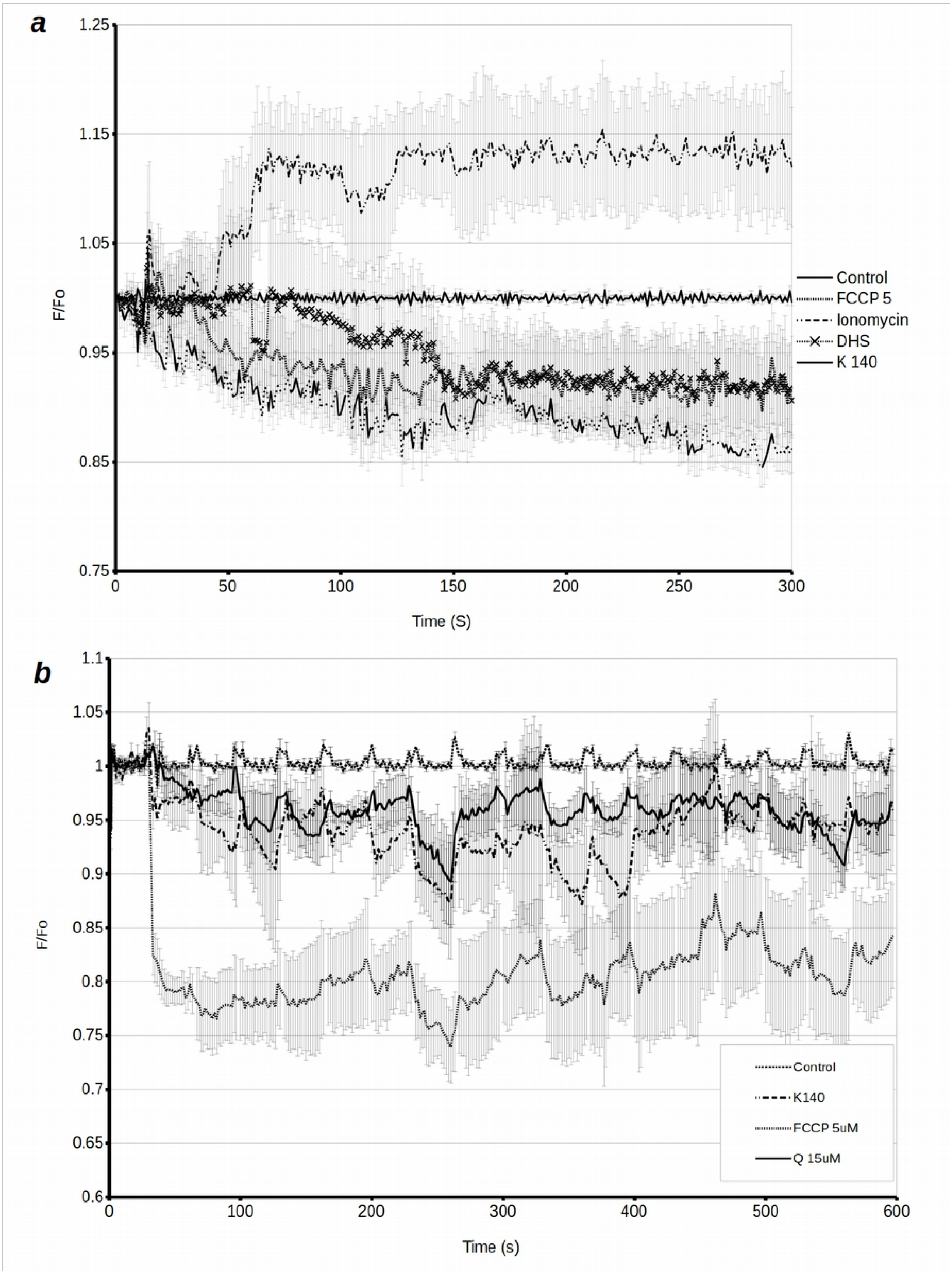

Fig B. Measurements of membrane potential using arclight plasmid. (Figure 7 + Error bars showing standard error of the mean). **a)** Measurements carried out using wide field microscopy for DHS. **b)** Measurements for quercetin, carried out using confocal microscopy.

OPEN DATA

The authors provide a minimal dataset in the form of CSV files. Below is an index of these files.

File	Figure
Fig2Supp(Oxygraphywhole).csv	Fig 2
Fig3Supp(Oxygraphypermeablised).csv	Fig 3
Fig4Supp(JC1).csv	Fig 4
FigSupp5(OxygraphyADP).csv	Fig 5
Fig6abSupp(OxygraphyAtr)	Fig 6a and 6b
Fig6c(BongkOxygraphy)	Fig 6c
Fig6dSupp(BongkLEAKOxygraphy)	Fig 6d
Fig7a(GECO)	Fig 7a
Fig7b(CEPIAhypoxia)	Fig 7b
Fig8aSupp(arclightwidefield)	Fig 8a
Fig8bSupp(arclightconfocal)	Fig 8b
Fig9aSupp(Fluo4QConfocal)	Fig 9a
Fig9bSupp(Fluo4DHSConfocal)	Fig 9b
Fig9cSupp(Fluo4QConfocal)	Fig 9c
Fig9dSupp(Fluo4NoCaDHSConfocal)	Fig 9d
Fig9eSupp(Fluo4Nif-Bay)	Fig 9e
Fig9fSupp(Fluo4MiRQConfocal)	Fig 9f
Fig9gSupp(Fluo4NoMiRDHSConfocal)	-

The data for figures 7 through to 9 comes from microscopy based time course experiments. The authors have not provided the raw micrographs here, but are willing to provide them upon request.
